# NOX4–TIM23 interaction regulates NOX4 mitochondrial import and metabolic reprogramming

**DOI:** 10.1016/j.jbc.2023.104695

**Published:** 2023-04-10

**Authors:** Jyotsana Pandey, Jennifer L. Larson-Casey, Mallikarjun H. Patil, Rutwij Joshi, Chun-sun Jiang, Yong Zhou, Chao He, A. Brent Carter

**Affiliations:** 1 Division of Pulmonary, Allergy, and Critical Care Medicine, Department of Medicine, University of Alabama at Birmingham, Birmingham, Alabama, USA; 2 Department of Medicine, Birmingham VAMC, Birmingham, Alabama, USA

**Keywords:** macrophage, mitochondria, NOX4, OPXPHOS, TIM23

## Abstract

Pulmonary fibrosis is a progressive lung disease characterized by macrophage activation. Asbestos-induced expression of nicotinamide adenine dinucleotide phosphate hydrogen oxidase 4 (NOX4) in lung macrophages mediates fibrotic progression by the generation of mitochondrial reactive oxygen species (ROS), modulating mitochondrial biogenesis, and promoting apoptosis resistance; however, the mechanism(s) by which NOX4 localizes to mitochondria during fibrosis is not known. Here, we show that NOX4 localized to the mitochondrial matrix following asbestos exposure in lung macrophages via direct interaction with TIM23. TIM23 and NOX4 interaction was found in lung macrophages from human subjects with asbestosis, while it was absent in mice harboring a conditional deletion of NOX4 in lung macrophages. This interaction was localized to the proximal transmembrane region of NOX4. Mechanistically, TIM23 augmented NOX4-induced mitochondrial ROS and metabolic reprogramming to oxidative phosphorylation. Silencing TIM23 decreased mitochondrial ROS and oxidative phosphorylation. These observations highlight the important role of the mitochondrial translocase TIM23 interaction with NOX4. Moreover, this interaction is required for mitochondrial redox signaling and metabolic reprogramming in lung macrophages.

Pulmonary fibrosis is a chronic fibrotic lung disease with high morbidity and mortality (1). Environmental exposure to asbestos is an important cause of pulmonary fibrosis. Although strict regulatory measures have been in place since the 1970’s, 1.3 million workers are exposed to hazardous levels of asbestos in the United States resulting in 100,000 deaths annually (2, 3). The incidence of asbestosis is increasing with no currently available treatment (4). Thus, understanding molecular mechanism(s) involved in the pathogenesis of asbestos-induced pulmonary fibrosis may lead to development of more effective therapies.

Macrophages contribute to asbestosis by producing profibrotic factors, collagen synthesis substrates, and generate high levels of mitochondrial reactive oxygen species (ROS) (5). Mitochondrial ROS production mediates the profibrotic polarization of lung macrophages to induce fibrotic remodeling (6, 7).

Nicotinamide adenine dinucleotide phosphate hydrogen (NADPH) oxidase 4 (NOX4) is an enzyme of the family NADPH oxidases. NOX4 is constitutively active having the primary function of ROS generation in the mitochondria. In the basal state, NOX4 resides in mitochondria in macrophages. NOX4 catalyzes the reduction of oxygen to superoxide anion-free radicals as well as H_2_O_2_ (8, 9). In asbestos-induced fibrosis, NOX4 regulates lung macrophage profibrotic polarization, mitochondrial biogenesis, and apoptosis resistance (10, 11). NOX4 also has a role in other murine models of pulmonary fibrosis (12). Although mitochondrial NOX4 has a critical role in asbestos-induced fibrosis, the mechanism(s) by which NOX4 localizes to the mitochondria in fibrosis is not known.

Here, we found that NOX4 is present in the mitochondrial matrix and that the import requires direct interaction with TIM23. Because one major role of NOX4 is the generation of mitochondrial ROS, silencing TIM23 abrogated NOX4-mediated ROS production in macrophages. We and others have shown that mitochondrial ROS induced metabolic reprogramming to oxidative phosphorylation by increasing transcriptional activation of PGC-1α (13, 14). Silencing TIM23 blocked NOX4 import into the mitochondrial matrix, decreased expression of PGC-1α, and reduced oxidative phosphorylation, suggesting that TIM23 regulates the role of NOX4 in metabolic reprogramming and regulating mitochondrial dynamics in asbestos-exposed macrophages.

## Results

### Asbestos-induced translocation of NOX4 to the mitochondrial matrix in macrophages

**Figure 1:**
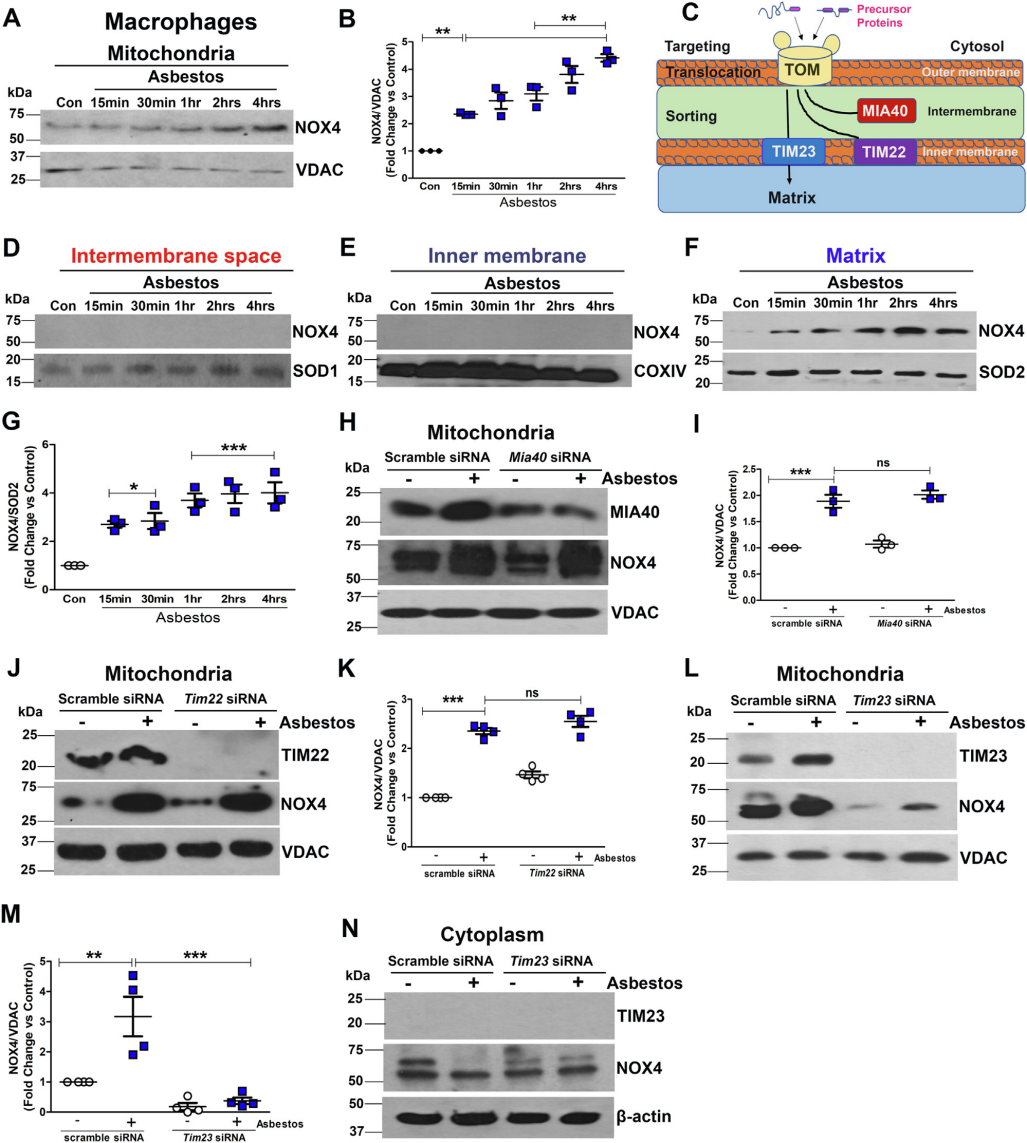
**Asbestos induces translocation of NOX4 to the mitochondrial matrix in macrophages.***A*, immunoblot analysis of NOX4 and VDAC in mitochondria isolated from macrophages. *B*, quantification of immunoblots shown in *A*. *C*, schematic showing mitochondrial protein translocation system. *D*, mitochondrial intermembrane space immunoblot analysis of NOX4 and SOD1. *E*, mitochondrial inner membrane immunoblots of NOX4 and COX IV. *F*, mitoplast (matrix) immunoblot analysis of NOX4 and SOD2. *G*, quantification of immunoblots from mitoplast. Macrophages were exposed to asbestos for 15 min, 30 min,1 h, 2 h, and 4 h of time (n = 3). *H*, immunoblot analysis in isolated mitochondria of MIA40, NOX4, and VDAC in transfected macrophages with scrambled and *Mia40* siRNA with and without asbestos exposure (n = 3). *I*, quantification of immunoblots shown in *G*. *J*, immunoblot analysis in isolated mitochondria of TIM22, NOX4, and VDAC in transfected macrophages with scrambled and *Tim22* siRNA with and without asbestos exposure (n = 4). *K*, quantification of immunoblots shown in *J*. *L*, immunoblot analysis in isolated mitochondria of NOX4, TIM23, and VDAC in transfected macrophages with scrambled and *Tim23* siRNA with and without asbestos exposure (n = 4). *M*, quantification of immunoblots shown in *L*. *N*, immunoblot analysis in isolated cytoplasm of NOX4, TIM23 and β-actin. One way ANOVA followed by Tukey’s *post-hoc* comparison ∗*p* < 0.05, ∗∗*p* < 0.01, ∗∗∗*p* < 0.001. NADPH, nicotinamide adenine dinucleotide phosphate hydrogen; NOX4, NADPH oxidase 4.

Asbestos increased NOX4 expression in mitochondria in a time-dependent fashion. (Fig.1, *A* and *B*). To determine the specific location of NOX4 in the macrophage mitochondria, we isolated intermembrane space (IMS), inner membrane (IM), and matrix (mitoplast, M) (Fig. 1*C*). NOX4 was not present in the IMS or IM (Fig. 1, *D* and *E*); however, NOX4 was localized in the matrix of macrophages exposed to asbestos over a prolonged time course (Fig. 1, *F* and *G*).

Mitochondrial protein translocation is a regulated process driven by various mitochondrial translocases (15). The outer membrane protein TOM is a universal import translocase that regulates movement of proteins into the mitochondria. MIA40, TIM22, and TIM23 are responsible for localization of proteins in the IMS, IM, and matrix. Asbestos induced an increase of NOX4 in the mitochondria, and silencing MIA40 had no effect on NOX4 expression (Fig. 1, *H* and *I*). Similar observations were found when TIM22 was silenced (Fig. 1, *J* and *K*). TIM23 is the translocase in the inner membrane that regulates the movement of proteins destined to mitochondrial matrix (16). Silencing TIM23 significantly reduced NOX4 expression in the mitochondria (Fig. 1, *L* and *M*). TIM23 is only found in the mitochondria and is not found in the cytoplasmic fraction (Fig. 1*N*).

### NOX4 overexpression augments colocalization with TIM23

The port of entry for mitochondrial proteins to move into the mitochondria is facilitated by TOM complex that directs the protein transport to specific mitochondrial compartments (17). We found colocalization of NOX4 with TOM20 in macrophages exposed to asbestos (Fig. S1, *B* and *C*). Overexpression of NOX4 had a similar increase in the localization.

**Figure 2:**
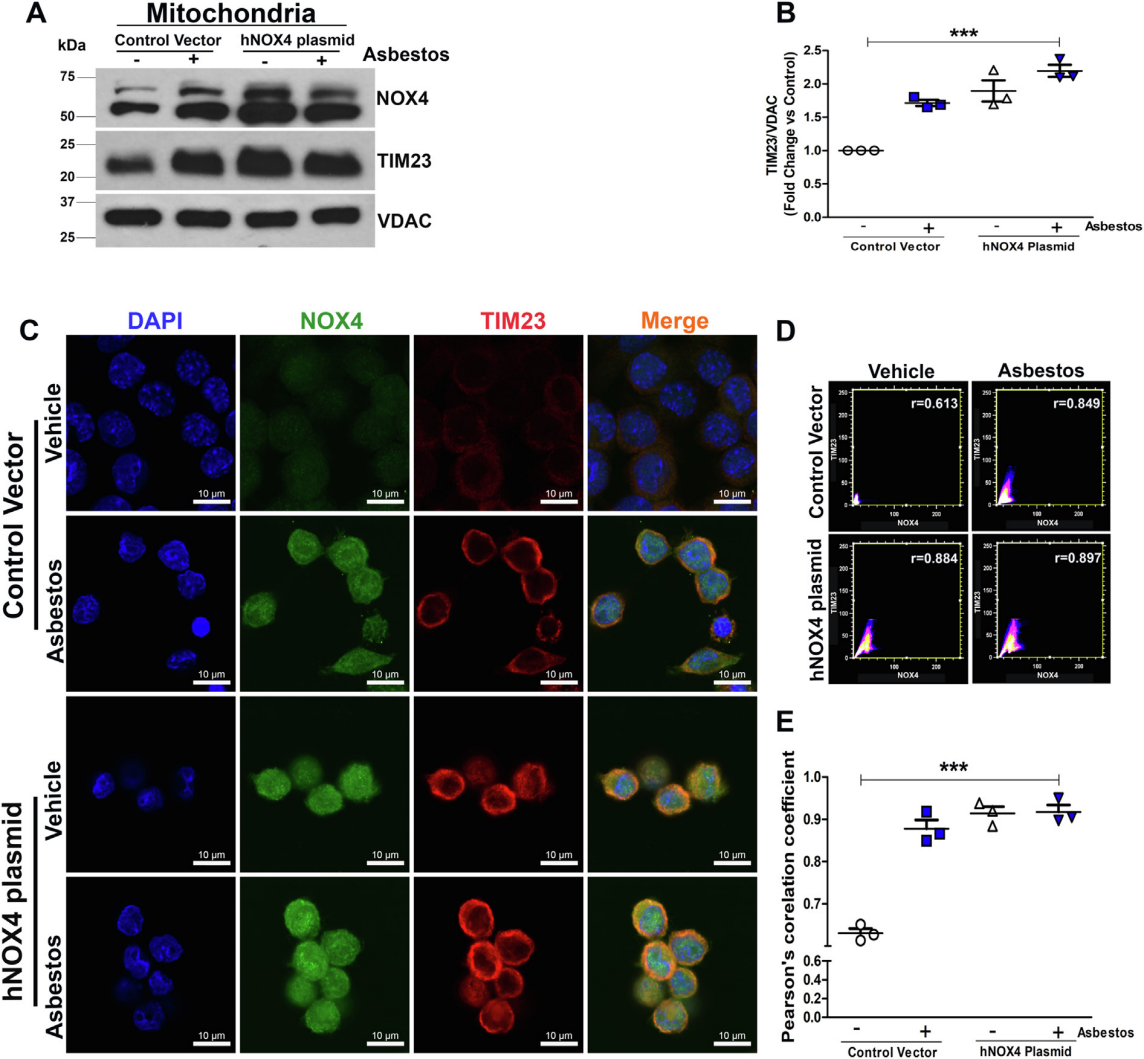
**NOX4 overexpression augments interaction with TIM23.***A*, immunoblot analysis in isolated mitochondria of NOX4, TIM23, and VDAC in transfected macrophages with empty vector and hNOX4 plasmid with and without asbestos (n = 3). *B*, quantification of immunoblots shown in *A*. *C*, confocal microscopy in transfected macrophages with empty vector and hNOX4 plasmid with and without asbestos exposure (n = 3). Representative images show NOX4 (*green*), TIM23 (*red*), and nuclei (*blue*), scale bar 10 μm. *D*, scatterplot indicate colocalization. *E*, Pearson’s correlation coefficient (n = 3). Data representative of mean (n = 3) ± S.E.M. One-way ANOVA with Tukey’s *post-hoc* comparison; ∗∗∗*p* < 0.001. NADPH, nicotinamide adenine dinucleotide phosphate hydrogen; NOX4, NADPH oxidase 4.

To determine if the colocalization of TIM23 and NOX4 was secondary to NOX4, we overexpressed NOX4. Asbestos increased both TIM23 and NOX4 (Fig. 2, *A* and *B*). Overexpression of NOX4 increased TIM23 to a similar level as asbestos exposure. To visualize this interaction, cells were stained with NOX4 and TIM23 antibodies. Colocalization of NOX4 with TIM23 occurred in asbestos-exposed macrophages. Overexpression of NOX4 increased the positioning of both proteins together in the presence or absence of asbestos (Fig. 2*C*). Asbestos exposure (Pearson’s *r* = 0.849) and NOX4 overexpression (Pearson’s *r* = 0.884 and *r* = 0.897) was highly correlated with TIM23 (Fig. 2, *D* and *E*).

### NOX4 and TIM23 localize in mitochondria in macrophages from asbestos-injured mice

**Figure 3:**
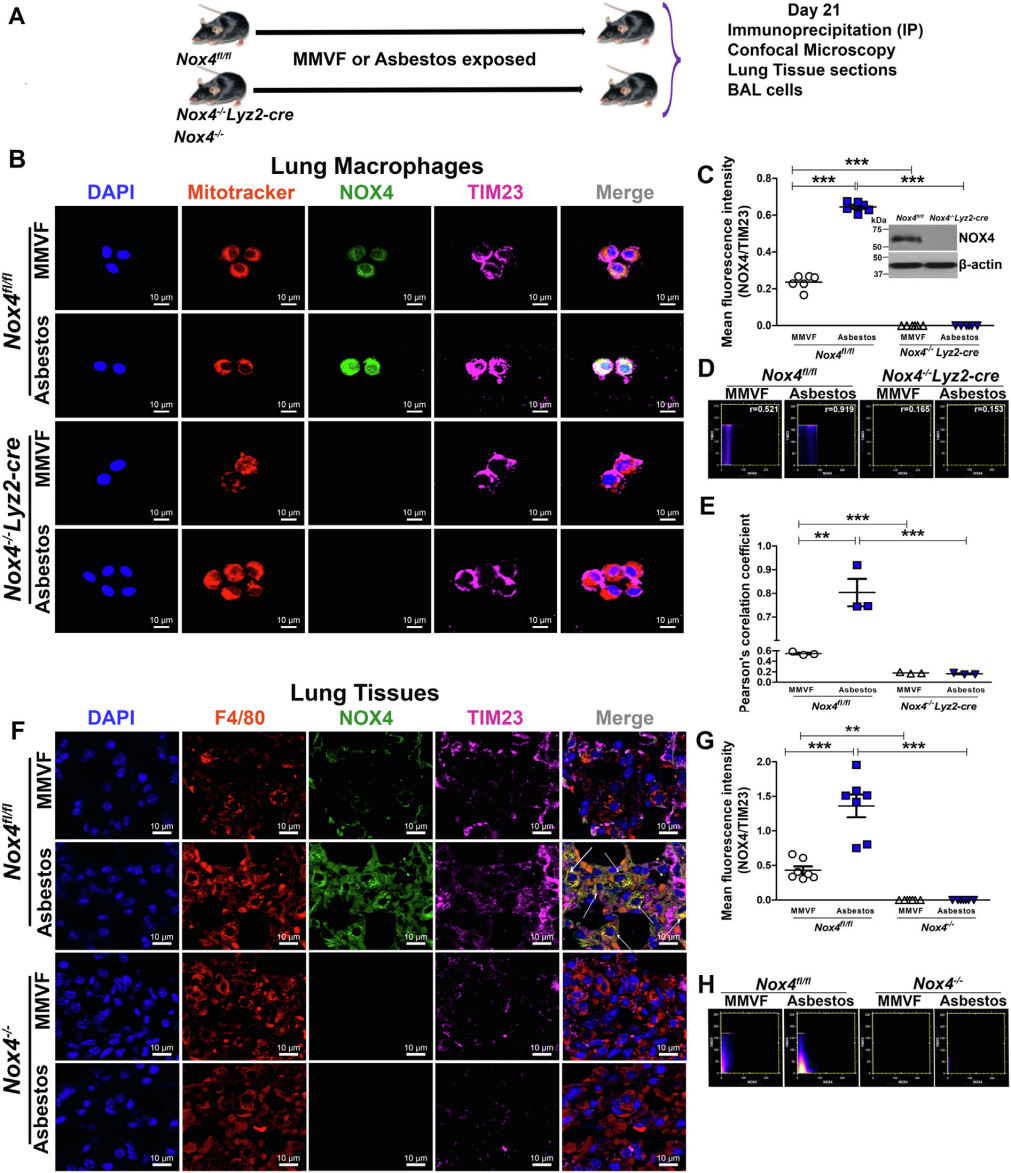
**NOX4 colocalizes with TIM23 in lung macrophage mitochondria from asbestos-injured mice.***A*, schematic plan of animal experiments. *B*, confocal microscopy in lung macrophage mitochondria of *Nox4*^*fl/fl*^ mice and macrophage-specific conditional knockout mice model of *Nox4*^*−/−*^*Lyz2-cre* with and without asbestos exposure (n = 3). Representative images show NOX4 (*green*), TIM23 (*pink*), Mitotracker (*red*), and nuclei (*blue*) scale bar 10 μm. *C*, bar graph for mean fluorescent intensity (n = 6). *D*, scatterplot indicate colocalization. *E*, Pearson’s correlation co-efficient (n = 3). *F*, confocal microscopy in macrophages of lung tissues sections from *Nox4*^*fl/fl*^ mice and *Nox4*^*−/−*^ with and without asbestos exposure (n = 3). Representative images show NOX4 (*green*), TIM23 (*pink*), F4/80 (*red*), and nuclei (*blue*) scale bar 10 μm. *G*, bar graph for mean fluorescent intensity of lung tissue sections (n = 7). *H*, scatterplot indicate colocalization. Data representative of mean (n = 3) ± S.E.M. One way ANOVA with Tukey’s *post-hoc* comparison; ∗∗*p* < 0.01, ∗∗∗*p* < 0.001. NADPH, nicotinamide adenine dinucleotide phosphate hydrogen; NOX4, NADPH oxidase 4.

We found that TIM23 is responsible for translocation of NOX4 protein to the mitochondrial matrix in macrophages in our *in vitro* experiments. To test if these findings were present *in vivo*, we utilized mice harboring a conditional deletion of *Nox4* in macrophages. *Nox4*^*fl/fl*^ and *Nox4*^*−/−*^*Lyz2-cre* mice were exposed to man-made vitreous fiber (MMVF) or chrysotile asbestos (Fig. 3*A*). At day 21, the percentage of macrophages in the BAL were greater than 90% of the cells in all mice (Fig. S1, *D* and *E*).

We questioned if NOX4 and TIM23 expression was regulated by transcription or secondary to protein degradation. We found that MG132 treatment does not alter the localization of NOX4 in mitochondria (Fig. S2*A*). Silencing NOX4 showed that asbestos did not increase TIM23 confirming that NOX4 is involved in transcriptional regulation of TIM23 (Fig. S2, *B* and *C*).

NOX4 and TIM23 showed significant colocalization in macrophage mitochondria with asbestos exposure in *Nox4*^*fl/fl*^ mice compared to the mice exposed to MMVF (Fig. 3, *B* and *C*). As expected, there was only TIM23 seen in the *Nox4*^*−/−*^*Lyz2-cre* mice. NOX4 was highly correlated with TIM23 in *Nox4*^*fl/fl*^ mice exposed to asbestos in macrophages (Pearson’s *r* = 0.919) (Figs. 3, *D* and *E* and S3).

To extend our findings in BAL macrophages, we questioned if there was localization of NOX4 and TIM23 in other macrophages in lung tissue. We found that NOX4 and TIM23 colocalized in lung macrophages in the *Nox*^*fl/fl*^ mice (Fig. 3, *F* and *G*). This was not seen in the *NOX4*^*−/−*^ mice. These observations validate that NOX4 and TIM23 colocalize in macrophage mitochondria from asbestos-exposed mice.

### NOX4 directly interacts with TIM23 in lung macrophages and mitochondria

**Figure 4:**
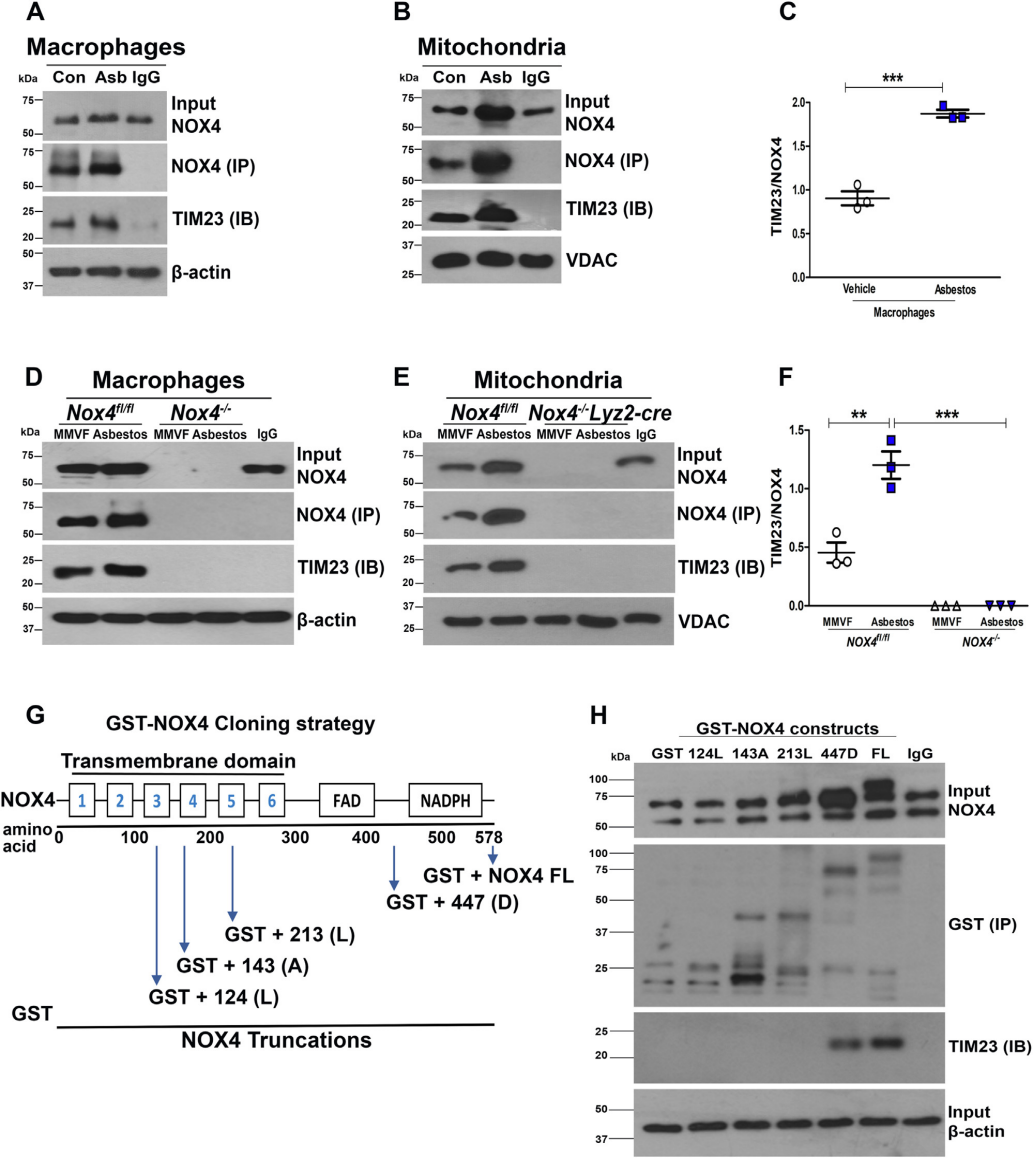
**NOX4 interacts with TIM23 in Lung macrophages and mitochondria.***A*, immunoblot analysis of immunoprecipitation in macrophages. *B*, immunoblot analysis of immunoprecipitation in isolated mitochondria from macrophages, showing interaction between NOX4 and TIM23. *C*, TIM23 was statistically quantified, n = 3. *t* test ∗∗∗*p* < 0.001. *D*, immunoblot analysis of immunoprecipitation in Nox4^*fl/fl*^ mice and Nox4^*−/−*^ mice in lung macrophages (n = 10). *E*, immunoblot analysis of immunoprecipitation in isolated mitochondria from lung macrophages of Nox4^*fl/fl*^ mice and Nox4^*−/−*^ Lyz2-Cre mice, showing interaction between NOX4 and TIM23 (n = 4 mice). *F*, TIM23 was statistically quantified, (n = 3). One-way ANOVA followed by Tukey’s post hoc comparison test ∗∗*p* < 0.01, ∗∗∗*p* < 0.001. *G*, schematic representation of NOX4-truncated mutant constructs. *H*, immunoblot analysis of immunoprecipitation for transfected GST-NOX4–truncated mutants and TIM23. NADPH, nicotinamide adenine dinucleotide phosphate hydrogen; NOX4, NADPH oxidase 4.

We questioned if there was a direct interaction between NOX4 and TIM23. Immunoblot analysis of TIM23 was present in cells lysates (Fig. 4*A*), and isolated mitochondria (Fig. 4, *B* and *C*) was subjected to NOX4 immunoprecipitation. Asbestos exposure increased TIM23 expression nearly 2-fold greater than control.

Asbestos-induced injury significantly increased NOX4-TIM23 direct interaction compared to the mice that were exposed to MMVF (Fig. 4, *D* and *F*). We confirmed that this interaction occurred in isolated mitochondria from asbestos-injured *Nox4*^*fl/fl*^ mice, whereas it was not present in the macrophage mitochondria from *Nox4*^*−/−*^*Lyz2-cre* mice (Fig. 4*E*)

NOX4 protein is composed of one transmembrane (TMB) domain, a FAD domain, and a NADPH domain. At the N terminus a 74 amino acid mitochondrial leader sequence (MLS) guides the protein to mitochondria, so we performed truncated mutations in the TMB domain instead of in the MLS. We cloned the GST-NOX4 mutant constructs that would correlate with amino acid residues at 124aa, 143aa, 213aa, 447aa, and NOX4 full-length mutation. The cloning construction was introduced at 372 nt, 429 nt, 639 nt, and 1341 nt, and NOX4 full-length protein is 1734 nt (Fig. 4*G*). There was direct interaction with the full-length GST-NOX4 and the GST-NOX4 447aa mutant, whereas TIM23 was absent with immunoprecipitation of all other constructs (Fig. 4*H*), suggesting that a putative motif from amino acids 213 to 447 are required for NOX4-TIM23 binding. In aggregate, these observations validate that NOX4-TIM23 direct interaction within the TMB region is required for NOX4 binding to TIM23 and the import into the mitochondria in lung macrophages from asbestos-exposed mice.

### NOX4 directly interacts with TIM23 in subjects with asbestosis

**Figure 5:**
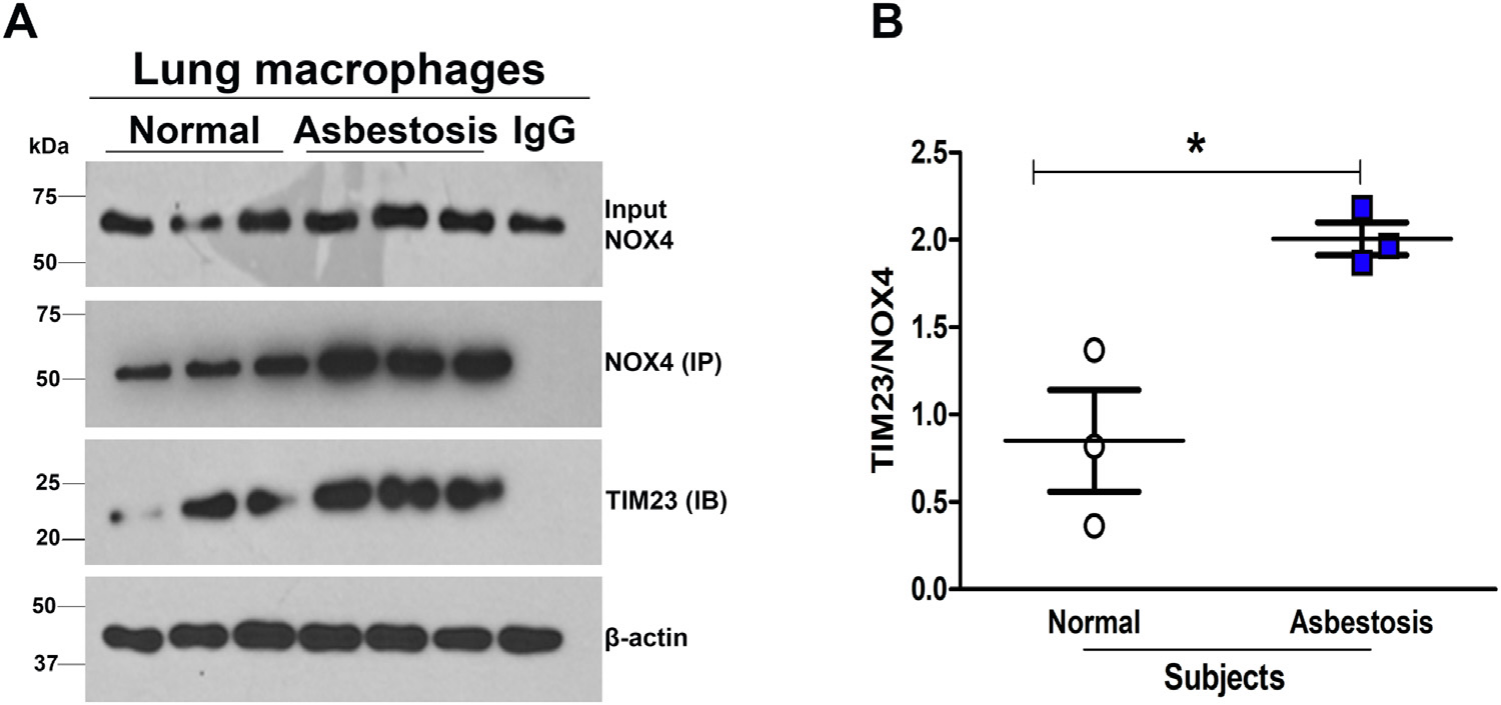
**NOX4 interacts with TIM23 in subjects with asbestosis.***A*, immunoblot of immunoprecipitation in normal or asbestosis subjects BAL macrophages showing interaction between NOX4 and TIM23. *B*, TIM23 was statistically quantified, n = 3. *t* test ∗*p* < 0.05; Healthy normal subjects compared to asbestosis subjects. NADPH, nicotinamide adenine dinucleotide phosphate hydrogen; NOX4, NADPH oxidase 4.

We have shown that lung macrophages from asbestosis subjects have increased mitochondrial NOX4 in addition to increased mitochondrial ROS production (10, 11). To provide evidence that NOX4–TIM23 interaction is biologically relevant in humans, we obtained lung macrophages from normal and asbestosis subjects. Lung macrophages were subjected to NOX4 immunoprecipitation, and immunoblot analysis showed that TIM23 was significantly increased in the lung macrophages from asbestosis subjects (Fig. 5, *A* and *B*). These observations are in support of our hypothesis that direct interaction of TIM23 and NOX4 is critical for the localization of NOX4 in mitochondria in humans subjects with asbestosis.

### NOX4 and TIM23 interaction mediate oxidative phosphorylation in lung macrophages

**Figure 6:**
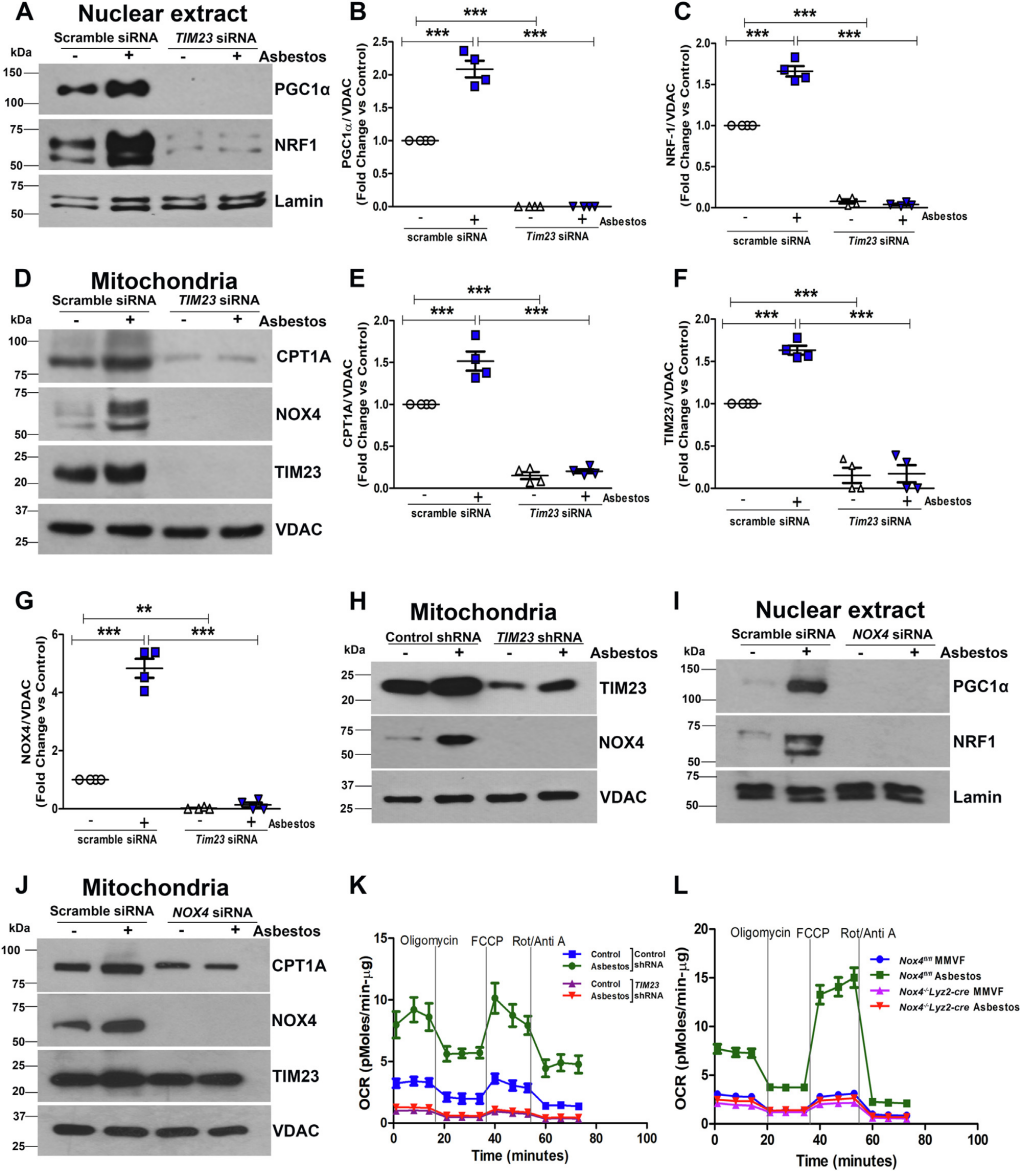
**NOX4 and TIM23 interaction mediates oxidative phosphorylation in lung macrophages.***A*, nuclear extract immunoblot analysis of PGC-1α, NRF-1, and lamin in transfected macrophages with scrambled and *Tim23* siRNA with and without asbestos exposure. *B*, quantification of immunoblots of PGC-1α. *C*, quantification of immunoblots of NRF-1. *D*, immunoblot analysis in isolated mitochondria of Cpt1A, NOX4, TIM23, and VDAC in transfected macrophages with scrambled and *Tim23* siRNA with and without asbestos exposure. *E*, quantification of immunoblots of Cpt1A. *F*, quantification of immunoblots of NOX4. *G*, quantification of immunoblots of TIM23. Data represented as (n = 4). *H*, immunoblot analysis in isolated mitochondria of transfected MH-S macrophages with scrambled and *Tim23* siRNA with and without asbestos exposure. *I*, nuclear extract immunoblot of PGC-1α, NRF-1, and lamin in transfected macrophages with scrambled and *Nox4* siRNA with and without asbestos exposure. *J*, immunoblot analysis in isolated mitochondria of Cpt1A, NOX4, TIM23, and VDAC in transfected macrophages with scrambled and *Nox4* siRNA with and without asbestos exposure. Data represented as (n = 3). *K*, oxygen consumption rate (OCR) tracing on the Seahorse XF96 bioanalyzer in transfected MH-S macrophages with scrambled and *Tim23* shRNA plasmid with and without asbestos exposure and subjected to OCR measurement (n = 5). *L*, OCR tracing on the Seahorse XF96 bioanalyzer in Nox4^*fl/fl*^ mice and Nox4^*−/−*^Lyz2-Cre mice exposed to MMVF or asbestos for 21 days. Lung macrophages from mice were subjected to OCR measurement (n = 3). Data representative of mean ± S.E.M. One-way ANOVA with Tukey’s post hoc comparison test, ∗∗*p* < 0.01; ∗∗∗*p* < 0.001. MMVF, manmade vitreous fiber; NADPH, nicotinamide adenine dinucleotide phosphate hydrogen; NOX4, NADPH oxidase 4.

Emerging data show a potential role of mitochondrial translocases in metabolic reprogramming (17, 18). We previously showed that increased NOX4 expression in lung macrophage mitochondria was linked to augmented mitochondrial biogenesis (10). These data suggest that mitochondrial NOX4 regulated a metabolic shift that contributed to activation of lung macrophages. To determine if NOX4-TIM23 interaction had a role in metabolic reprogramming, we found that asbestos-exposed macrophages had a significant increase in peroxisome proliferator-activated receptor-gamma coactivator-1apha (PGC-1α) and nuclear respiratory factor 1 (NRF1) in macrophage nuclei, whereas there was an absence of PGC-1α and greatly reduced NRF1 when TIM23 was silenced (Fig. 6, *A*–*C*) Because macrophages with profibrotic polarization have increased CPT1A and metabolic reprogramming to fatty acid oxidation (19), we next questioned if NOX4–TIM23 interaction regulated carnitine palmitoyltransferase 1A (Cpt1a) expression. Asbestos exposure increased Cpt1a, NOX4, and TIM23 expression in isolated mitochondria (Fig. 6, *D*–*G*) In contrast, Cpt1a and NOX4 were significantly reduced when TIM23 was silenced. Similar results were found in the nuclear and mitochondrial fractions of asbestos-exposed macrophages when NOX4 was silenced (Fig. 6, *H*–*J*).

Because PGC-1α increases the enzymatic capacity of oxidative phosphorylation, we asked if NOX4–TIM23 interaction influenced the oxidative consumption rate (OCR). Asbestos increased OCR in cells transfected with the control shRNA, whereas OCR was significantly reduced when TIM23 was silenced (Fig. 6*K*). OCR in lung macrophages from asbestos-exposed *Nox4*^*fl/fl*^ mice was significantly increased, while *Nox4*^*−/−*^*Lyz2-Cre* mice exposed to MMVF or asbestos for 21 days were at the MMVF control level (Figs. 6*L* and S4, *A* and *B*). Taken together, these observations strongly suggest that the direct interaction of NOX4 with TIM23 is necessary for metabolic reprogramming to oxidative phosphorylation in lung macrophages (Fig. S5).

### TIM23 and NOX4 interaction mediates mitochondrial ROS and mitochondrial dynamics in macrophages

**Figure 7:**
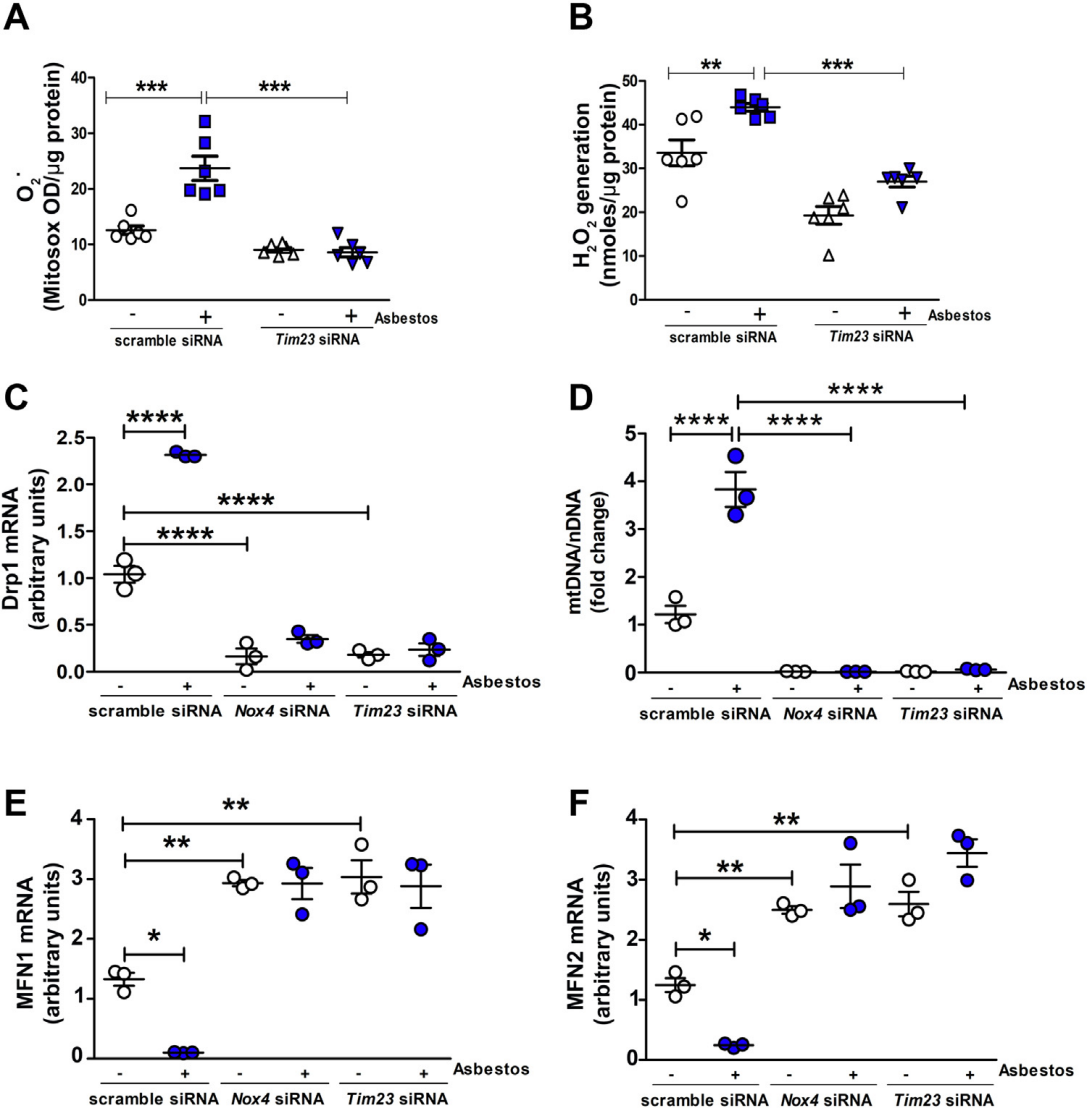
**TIM23 and NOX4 interaction mediates mitochondrial ROS and mitochondrial dynamics in macrophage.***A*, mitosox assay in transfected macrophages with scrambled and *Tim23* siRNA with and without asbestos exposure for mitochondrial superoxide determination. *B*, pHPA assay in transfected macrophages with scrambled and *Tim23* siRNA with and without asbestos exposure for mitochondrial hydrogen peroxide (n = 6). *C*, Drp1 mRNA analysis in transfected macrophages with scrambled and *Nox4* siRNA and *Tim23* siRNA with and without asbestos exposure (n = 3). *D*, mtDNA/nDNA analysis in transfected macrophages with scrambled and siRNA *Nox4* and siRNA *Tim23* with and without asbestos exposure (n = 3). *E*, Mnf1 mRNA analysis in transfected macrophages with scrambled and *Nox4* siRNA and *Tim23* siRNA with and without asbestos exposure (n = 3). *F*, Mnf2 mRNA analysis in transfected macrophages with scrambled and *Nox4* siRNA and *Tim23* siRNA with and without asbestos exposure (n = 3). Data representative of mean (n = 3) ± S.E.M. One-way ANOVA with Tukey’s post hoc comparison test, ∗*p* < 0.05, ∗∗*p* < 0.01, ∗∗∗*p* < 0.001, ∗∗∗∗*p* < 0.0001. NADPH, nicotinamide adenine dinucleotide phosphate hydrogen; NOX4, NADPH oxidase 4; ROS, reactive oxygen species.

ROS from the mitochondria are known to have an important role in pulmonary fibrosis in multiple cells, including lung macrophages (10, 20, 21, 22, 23, 24). To determine if interaction of TIM23 with NOX4 had a role in generation of ROS, we found that silencing TIM23 significantly reduced asbestos-induced mitochondrial O_2_^.-^ and H_2_O_2_ production in macrophages (Fig. 7, *A* and *B*).

We asked what the impact of NOX4 and TIM23 interaction had on mitochondrial dynamics. We found that Drp1 was reduced when NOX4 or TIM23 was silenced, whereas asbestos significantly increased Drp1 expression in cells with the scrambled siRNA (Fig. 7*C*). The mitochondrial to nuclear DNA ratio was significantly reduced in cells with NOX4 or TIM23 silenced (Fig. 7*F*). In cells transfected with the scrambled siRNA, asbestos markedly increased mDNA/nDNA.

Expression of mitochondrial fusion proteins, Mitofusin1 and Mitofusin2, was significantly increased when NOX4 or TIM23 was silenced, whereas asbestos-exposed macrophages with the scrambled siRNA significantly reduced Mitofusin1 and Mitofusin2 (Fig. 7, *D* and *E*). Taken together, these data indicate that NOX4 interacts with TIM23 to enter the mitochondrial matrix, which is required for mitochondrial ROS production. NOX4 transcriptionally regulates TIM23, and this interaction regulates mitochondrial dynamics, specifically biogenesis.

## Discussion

Asbestos-induced pulmonary fibrosis is a progressive, irreversible lung disease associated with high mortality (25). NOX4 is known to induce fibrotic diseases in liver (26), heart (27), and lung (10, 28, 29). NOX4 upregulation in the myocardium causes cardiac remodeling *via* activation of Akt-mTOR and NF-κB signaling pathways (30). NOX4 is found to be involved in activation of myofibroblasts followed by progression of hepatic fibrosis (31). Others reported that silencing NOX4 in fibroblasts attenuated bleomycin-induced pulmonary fibrosis (12). Our group previously reported that mice harboring a conditional deletion of NOX4 in macrophages were protected from asbestos-induced pulmonary fibrosis (10). Moreover, established fibrosis resolves with the conditional deletion of NOX4 in macrophages (11). In aggregate, these studies suggest that mitochondrial NOX4 has a critical role in multiple cell types that have a role in fibrotic remodeling.

Because 99% of mitochondrial proteins are synthesized on cytosolic ribosomes (32), mitochondrial translocases are key players in translocation of proteins into specific locations in the mitochondria. Proteins destined to the mitochondrial matrix have a mitochondrial localization sequence for the TOM–TIM23 complex to mediate the translocation (33). The importance of TIM23 was shown in an animal model of Huntington’s disease. The mutated huntingtin protein prevented mitochondrial import, while overexpression of TIM23 restored mitochondrial import and prevented neuronal death (34). Furthermore, lifespan in TIM23 knockout mice is reduced, suggesting the importance of TIM23-mediated mitochondrial import for long-term survival (35). The MLS is the N-terminal segment of preproteins necessary for the coordinated action of TOM and TIM complexes for translocation of proteins (36). The MLS of NOX4 is amino acids 1 to 74 (37). Our data showed that NOX4 interacts with TOM and TIM23 for the import of NOX4 into the mitochondrial matrix in asbestos-exposed macrophages. Importantly, the direct interaction of NOX4 with TIM23 in the transmembrane domain is required for the profibrotic function of NOX4.

NOX4 is highly expressed in macrophages in conditions associated to oxidative stress. We have shown that subjects with asbestosis have increase transcription of *Nox4* compared to normal subjects (10). Furthermore, asbestosis subjects have increased localization of NOX4 in mitochondria suggesting that there is rapid translocation into the mitochondria. Our current data support this as there is an increase in expression of NOX4 in mitochondria within 15 min after asbestos exposure making expression in the cytoplasm reduced to absent. Splice variants of NOX4 occur in some cells (38), but two variants have reduced ROS generation. The other two splice variants lack TMB domains. Our data clearly demonstrate that these variants do not exist in macrophages; however, increased transcription does occur in subjects with asbestosis.

Reduction of NOX4-mediated ROS production was protective in various diseases, including cardiac ischemia/reperfusion injuries, diabetes, and *Streptococcus pneumonia* infection as well as fibrotic progression (39, 40). NOX4-induced ROS production was also found to be involved in various oncogenic signaling pathways in cancer (41). We found that reduction of TIM23–NOX4 interaction abrogates mitochondrial ROS and metabolic reprogramming, suggesting that TIM23–NOX4 interaction may become an important therapeutic target for the treatment of lung fibrosis.

NOX4 regulates metabolic reprogramming in various diseases, such as cardiac hypertrophy (42), cancers (43), and pneumonia (44). In thyroid carcinoma, NOX4 induced glycolysis by shifting oxidative phosphorylation to glycolysis (45) Another study showed that NOX4 deletion suppresses the aerobic respiration in glioblastoma (43). Deficiency of NOX4 also reduced expression of Cpt1a (40), whereas increased NOX4 mediated fatty acid oxidation and a reduction in glucose utilization in the heart (42). Our data demonstrated that asbestos and NOX4 induced metabolic reprogramming to oxidative phosphorylation.

Although we have shown that NOX4 regulates mitochondrial biogenesis (10), the new findings here show the interaction of NOX4 with TIM23 is required to mediate fission and biogenesis. NOX4 has a direct role by increasing TIM23 transcription. During fibrosis, we have found that mitochondrial biogenesis is necessary for apoptosis resistance in lung macrophages, and NOX4 has a role in this, as well (11, 46).

Taken together, these observations revealed that the direct interaction of TIM23 and NOX4 is crucial for the function of NOX4. This direct interaction results in generation of mitochondrial ROS, metabolic reprogramming, and mitochondrial dynamics in macrophages, all of which are important in development and progression of lung fibrosis. Our results further suggest that this interaction may be a druggable target for asbestosis and other fibrotic lung diseases.

## Experimental procedures

### Human subjects

Human BAL cells were obtained as previously described (20) from normal subjects and asbestosis subjects under an approved protocols by the Human Subjects Institutional Review Boards of UAB (300001124) and the Birmingham VAMC (01670). All studies follow the Declaration of Helsinki principles, and human BAL samples were used for research only. All subjects provided prior written consent to participate in the study. Normal volunteers had to meet the following criteria: (1) age between 18 and 75 years; (2) no history of cardiopulmonary disease or other chronic disease; (3) no prescription or nonprescription medication except oral contraceptives; (4) no recent or current evidence of infection; and (5) lifetime nonsmoker. Asbestosis subjects had to meet the following criteria: (1) forced vital capacity at least 50% predicted; (2) current nonsmoker; (3) no recent or current evidence of infection; (4) evidence of restrictive physiology on pulmonary function tests; (5) usual interstitial pneumonia on high-resolution chest computed tomography; and (6) have occupational or environmental exposure to asbestos. Fiberoptic bronchoscopy with bronchoalveolar lavage was performed after subjects received local anesthesia. Three subsegments of the lung were lavaged with five 20-ml aliquots of normal saline, and the first aliquot in each was discarded. The percentage of macrophages was determined by Wright Giemsa stain and varied from 90 to 98%.

### Mice

UAB Institutional Animal Care and Use Committee approved the animal experiments (21149). All the experiments were performed as per NIH guidelines. *Nox4*^*−/−*^ and *Nox4*^*−/−*^*Lyz2-cre* mice have been previously described (10). Six to twelve-week-old male and female mice were intratracheally administered with 100 μg of chrysotile asbestos or MMVF, as control, suspended in 50 μl 0.9% saline solution after being anesthetized with 3% isoflurane using a precision Fortec vaporizer (Cyprane). On day 21, BAL was performed, and lung tissues were extracted.

### Cell culture and asbestos exposure

Mouse alveolar macrophage and human monocyte (THP-1) cell lines were obtained from ATCC. Cells were maintained in RPMI-1640 medium (Thermo Fisher Scientific) with the following supplements: 10% fetal bovine serum and penicillin/streptomycin. All experiments were performed with 0.5% serum supplement (11). 10 μg/cm^2^ asbestos was used to expose the cells *in vitro*, as previously described (47).

### Plasmids, transfections, siRNA, and shRNA

The pcDNA3.1-hNOX4, pcDNA3 C-terminal GST pBSU6-shTIM23/CMV-eGFP, and pBSU6-shScr/CMV-eGFP were purchased from Addgene. Plasmids were transfected with X-tremeGene nine Transfection Reagent (Roche# 06365809001), according to the manufacturer’s protocol. After 24 to 72 h, cells were subjected to expose to vehicle or asbestos followed by collection for downstream purposes. All siRNA were purchased from Integrated DNA Technologies. Cells were transfected using Dharma-FECT 4 (Dharmacon# T-2004) or DharmaFECT 2 (Dharmacon# T-2002), according to the manufacturer’s protocol. Eight hours following transfection, media were replaced, and cells were allowed to recover for 24 to 72 h.

Full-length and truncated human Nox4 cDNA were PCR amplified from pcDNA3.1-hNOX4 vector using primer sequence; hNox4 full length, 1734 nt: AAGCTTGGTACCGAGCTCGGATCCGCCACCATGGCTGTGTCC and CTTATCATCATCATCAACGGATCCGCTGAAAGACTCTTTATTG; hNox4 372 nt: CTTATCATCATCATCAACGGATCCCAGATGGGCAGCCACATG, hNox4 429 nt: CTTATCATCATCATCAACGGATCCTGCATTCAGTTCAACAAAG; hNox4 1-639 nt: CTTATCATCATCATCAACGGATCCCAGCAGCCCTCCTGAAAC; hNox4 1-1341 nt: CTTATCATCATCATCAACGGATCCATCCAACAGGGTGTTGAG. The PCR products were cloned into BamH1 digested pcDNA3-c-terminal GST vector by Gibson Assembly kit (Catalog No E5510S, New England Biolabs) as company’s instruction. The correct full-length or truncated hNox4 sequences were confirmed by DNA sequencing.

### Isolation of mitochondria, cytoplasm, intermembrane space, inner membrane, and nuclei

Mitochondria and nuclei were isolated as previously described (6). Briefly, mitochondria were isolated by lysing the cells in mitochondria buffer I containing 10 mM Tris, pH 7.4 0.2 mM EDTA, 320 mM sucrose, and protease inhibitors. Lysates were homogenized using a Kontes Pellet Pestle Motor and centrifuged at 2000*g* for 10 min at 4 °C. The supernatant was collected and kept at 4 °C, while the pellet was lysed, homogenized, and centrifuged again in similar manner. The two supernatants were pooled together and centrifuged at 12,000*g* for 15 min at 4 °C. The pellet was the mitochondrial fraction, and supernatant was the cytoplasmic fraction. The pellet was washed twice in the mitochondrial buffer I and resuspended in mitochondria buffer II without sucrose. Mitochondria was used for mitochondrial protein analysis.

Isolated mitochondria are subjected to isolation of inner mitochondrial compartments according to protocol (20, 48). Briefly for intermembrane isolation, mitochondrial fractions were treated with digitonin (0.1 mg of digitonin/mg of mitochondria) at room temperature for 1 h followed by centrifugation at 10,000*g* for 10 min. The supernatant was collected as intermembrane space fraction, and the pellet was collected for further inner membrane isolation according to protocol (49). Pellet was dissolved in mitochondrial buffer I with sucrose followed by sonication on ice. Samples were ultracentrifuged at 100,000*g*, and the pellet was collected as inner membrane fraction and dissolved in mitochondrial buffer II. To isolate the matrix (mitoplast), isolated mitochondria were incubated in a 1:5 volume of ice-cold hypotonic solution containing 10 mM Tris, pH 7.4, 1 mM EDTA, and 1 mM dithiothreitol and incubated for 10 min on ice. 150 mM NaCl was further added to the buffer for another 10 min on ice. Samples were centrifuged at 18,000*g* for 20 min at 4 °C, and the pellet was collected as mitochondrial matrix and dissolved in mitochondrial buffer II.

Nuclear isolation was performed by resuspending cells in a lysis buffer containing 10 mM Hepes, 10 mM KCl, 2 mM MgCl_2_, 2 mM EDTA, and keep on ice for 15 min. Nonidet P-40 (10%) was added to lyse the cells, and the cells were centrifuged at 14,000 rpm at 4 °C. The nuclear pellet was resuspended in an extraction buffer containing 50 mm Hepes, 50 mM KCl, 300 mM NaCl, 0.1 mM EDTA, and 10% glycerol for 20 min on ice followed by centrifuging at 14,000 rpm at 4 °C. The final supernatant was collected as nuclear extract.

### Determination of ROS generation

O_2_^-.^ and H_2_O_2_ production was determined fluorometrically as described previously (21).

### Immunoblot analysis

Immunoblot analysis was performed as previously described (50). Primary antibodies used were as follows: NOX4 rabbit polyclonal Ab and Tom20 anti-mouse Ab (H00009804-M01) were from Novus Biologicals (NB110-58849). CHCHD4 (MIA40) rabbit polyclonal Ab (21090-AP), TIM23 anti-mouse Ab (67535-1-Ig), TIM23 anti-rabbit Ab (11123-1-AP), and TIM22 anti-mouse Ab (14927-1-AP) were from Proteintech. NOX4 rabbit polyclonal Ab was from Abcam (ab109225). SOD1 anti-sheep Ab (07-403-I) and SOD2 anti-rabbit Ab (06-984) were from Millipore Sigma. PGC-1α anti-rabbit Ab (2178S), VDAC anti-rabbit Ab (4866), Lamin A/C anti-rabbit Ab (2032), NRF1 anti-rabbit Ab (12381S), COX IV anti-mouse Ab (11967S), and GST anti-mouse Ab (2624) were from Cell Signaling Technology, and GST (Z-5) (sc-459) rabbit Ab was from Santa Cruz Biotechnology. Densitometry was performed using Image J software.

### Confocal imaging

Fluorescence staining protocol was described previously (7, 19). Briefly macrophages and BAL cells were fixed with 4% paraformaldehyde at room temperature for 45 min followed by permeabilization for 5 min in ice-cold buffer (0.1% sodium citrate and 0.1% Triton X-100 in distilled water). Cells were blocked at room temperature for 1 h in Dulbecco's phosphate-buffered saline with 10% bovin serum albumin (BSA) and 10% goat serum and then incubated with TruStain FcX PLUS anti-mouse CD16/32 (BioLegend;156604) to block nonspecific binding of immunoglobulin to the Fc receptors. Further incubations were performed with TIM23 anti-mouse Ab (67535-1-Ig), NOX4 anti-rabbit polyclonal Ab (NB110-58849), F4/80-PE anti-mouse (BioLegend; 123109), MitoTracker Red (Thermos fisher Scientific; M7512), Goat Anti-Rabbit IgG-FITC (Southern Biotech; 4030-02), Goat Anti-Mouse IgG –TRITC (Southern Biotech; 1030-03), and Goat Anti-Mouse IgG1-AF647 (Southern Biotech; 1073-31) for 1 h with 2% BSA and 2% Goat serum BSA in each antibody for staining. DAPI (MP Biologicals; 157577) is used for nuclear staining for 10 min. Mitotracker red is used for mitochondrial staining for 20 min. Cells were fixed with cover slide using ProLong Gold Antifade Mountant (Thermo Fisher Scientific; P36930) followed by confocal microscopy. The Nikon A1 confocal microscope was used for imaging, and all the images were quantitated using ImageJ (NIH).

### Immunohistochemistry

Immunohistochemistry protocol was previously described (7). Briefly, lung tissue sections from mouse (4-μm-thick) were prepared and deparaffinized by incubating at 60 °C for 30 min and 2X wash in xylene for 5 min each. Tissues were rehydrated with gradient series of ethanol (absolute; 95%, 90%, 80%, and 70% in water) with 3 min each incubation followed by blocking in PBS containing 10% BSA and 10% normal goat serum then incubated with TruStain FcX PLUS anti-mouse CD16/32 (BioLegend;156604) to block nonspecific binding of immunoglobulin to the Fc receptors. All the sections were stained with NOX4 anti-rabbit polyclonal Ab (NB110-58849), TIM23 anti-mouse Ab (67535-1-Ig), F4/80-PE anti-mouse (BioLegend; 123109), Goat Anti-Rabbit IgG-FITC (Southern Biotech; 4030-02), and Goat Anti-Mouse IgG1-AF647 (Southern Biotech; 1073-31) in 2% BSA and 2% normal goat serum for 1 h each and then counterstained with DAPI (MP Biologicals; 157577). Tissue sections were fixed with cover slide using Vecta Mount, permanent mounting medium (Vector Laboratories; H-5000), followed by confocal microscopy. The Nikon A1 confocal microscope was used for imaging, and all the images were quantitated using ImageJ (NIH).

### OCR measurement

OCR measurement by a Seahorse XF96 bioanalyzer (Seahorse Bioscience) was performed as described (19). OCR measurement is performed in macrophages and BAL macrophages from *Nox4*^*fl/fl*^ mice and *Nox4*^*−/−*^*Lyz2-cre* mice exposed to MMVF or asbestos for 21 days.

### Immunoprecipitation

Immunoprecipitation was performed as previously described (19). Equal amounts of total protein from each supernatant were incubated overnight with the beads–antibody complex at 4 °C. The isolated mitochondria were treated with digitonin first followed by sonication before incubation the beads–antibody complex. The incubated complex was then washed three times followed by elution and immunoblot analysis.

### Quantification of mitochondrial DNA

The ratio of mitochondrial to nuclear DNA was determined as previously described (46).

### Quantitative real-time PCR

Total RNA was isolated from macrophages reverse transcribe, and quantitative real-time PCR was performed as described previously (20). Data were calculated by the cycle threshold (ΔΔCT) method, normalized to HPRT, and expressed in arbitrary units. The following primer sets were used: human Drp-1: 5′-GCT CCA GGA CGT CTT CAA CA-3' and 5′-TCT GCT TCC ACC CCA TTT TCT-3'; human HPRT: 5′-AGC CCT GGC GTC GTG ATT AGT GA-3' and 5′-AGC CCT GGC GTC GTG ATT AGT GA-3'; human MFN1: 5′-GCT GTT GCC GGG TGA TAG TTG -3′and 5′-TAG CCA GCA CAA AGT GCT TCA G -3’; and human MFN2: 5′-CCG GGA AGG TGA AGC GCA ATG-3′ and 5′-CTT CAA GGA AGG TGG CGC TCT-3’; human TIM23 : 5′-CGATACCTCGTGCAGGATACA-3' and 5′-GTCAGACCACCTCGTGCTAT-3'; human NOX4: 5′-CGT CTG GGC AGC TGA GTG -3' and 5′-GAG CCA GAT GAA CAG GCA GA-3'.

### Statistical analysis

Statistical comparisons were performed using one-way ANOVA with a Tukey’s post hoc test or *t* test. The statistical analysis was expressed as ± SEM and *p* < 0.05 being significant. GraphPad Prism statistical software was used for all analysis.

## Data availability

All the data in this manuscript are compiled and saved in “Carter Lab” on UAB server.

## Supporting information

This article contains supporting information.

## Conflict of interest

The authors that they have no conflicts of interest with the contents of this article.
