# Supplementary figures and images for: NOX4–TIM23 interaction regulates NOX4 mitochondrial import and metabolic reprogramming
Source: J Biol Chem. 2023 Apr 10;299(5):104695. doi: 10.1016/j.jbc.2023.104695 (PMC10193017; doi:10.1016/j.jbc.2023.104695)

# Supplementary Figure 1

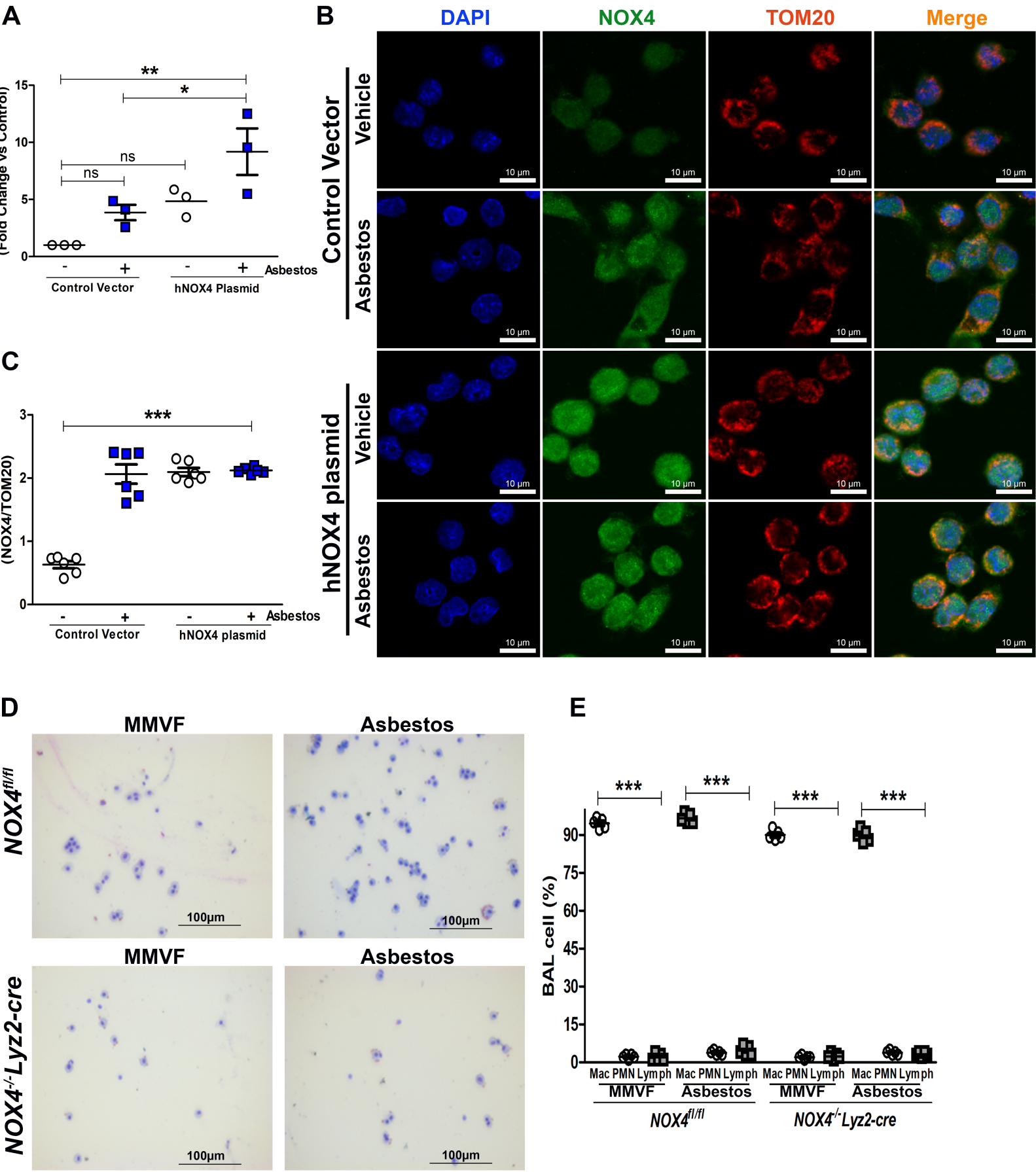

Supplement: Supporting Figure S1 [file mmc1.pdf]

# Supplementary Figure 2

A

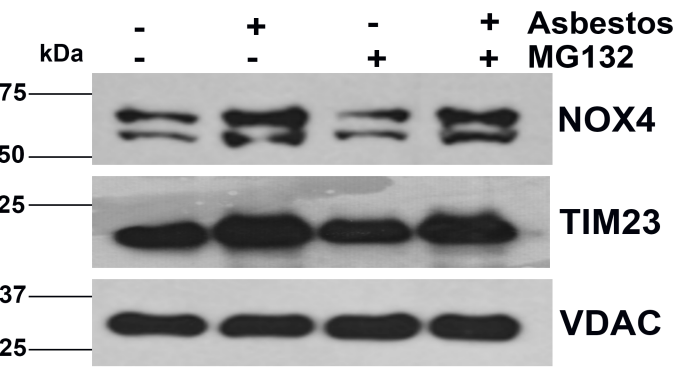

B

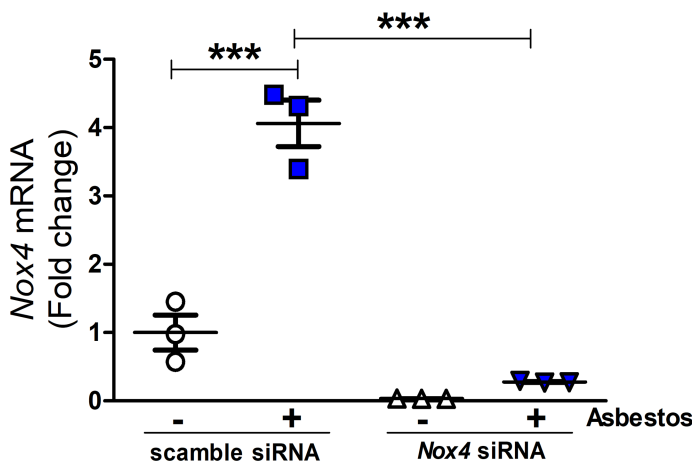

C

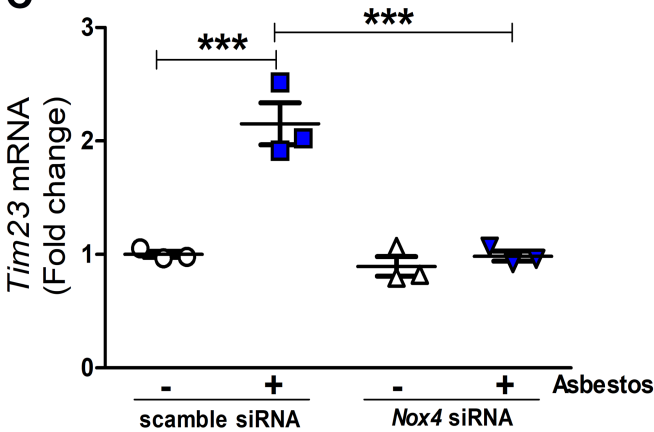

Supplement: Supporting Figure S2 [file mmc2.pdf]

# Supplementary Figure 4

A

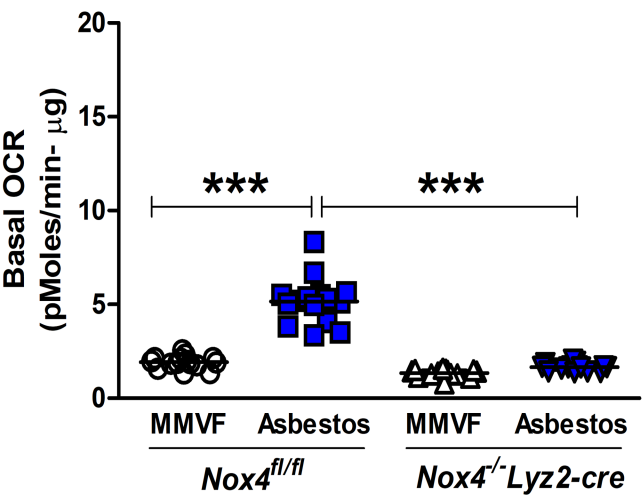

B

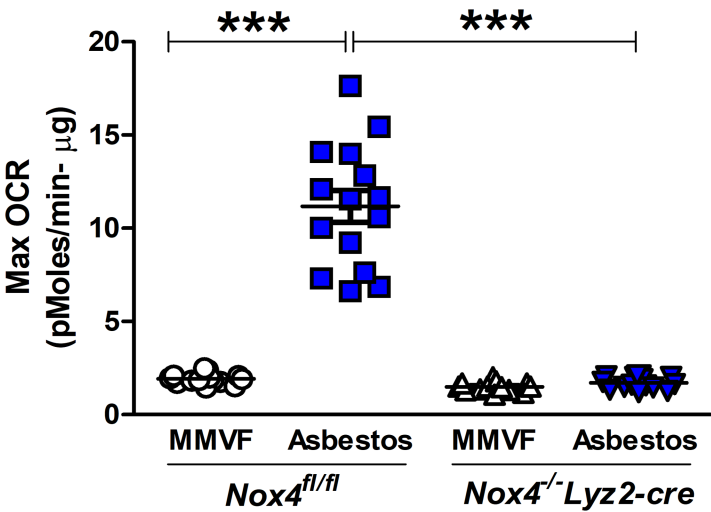

Supplement: Supporting Figure S4 [file mmc4.pdf]

Supplementary Figure 5

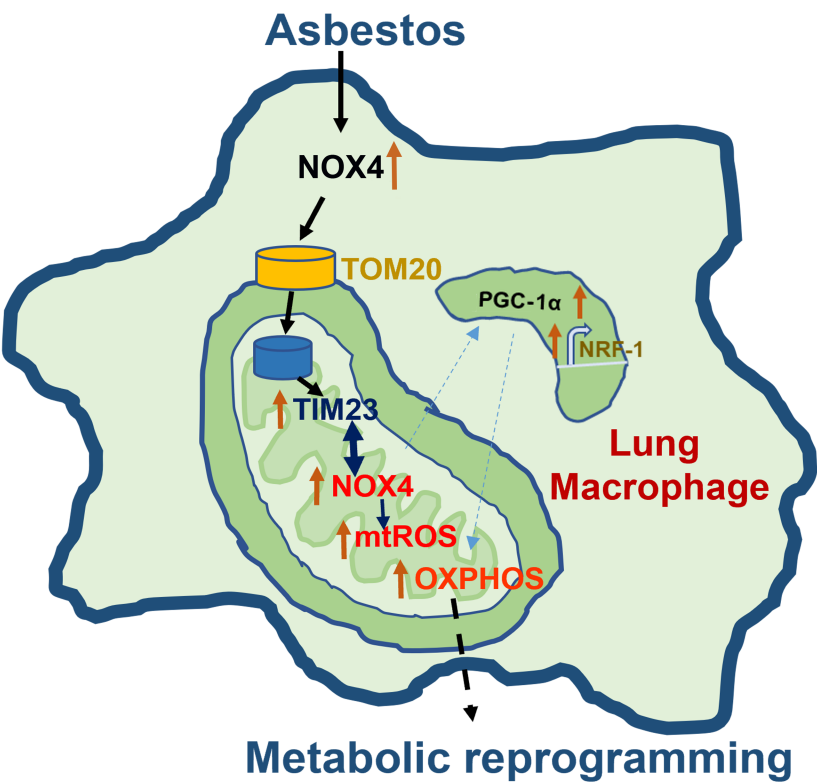

Supplement: Supporting Figure S5 [file mmc5.pdf]
