# Supplementary material for: NOX4–TIM23 interaction regulates NOX4 mitochondrial import and metabolic reprogramming
Source: J Biol Chem. 2023 Apr 10;299(5):104695. doi: 10.1016/j.jbc.2023.104695 (PMC10193017; doi:10.1016/j.jbc.2023.104695)

Supplementary Figure 3

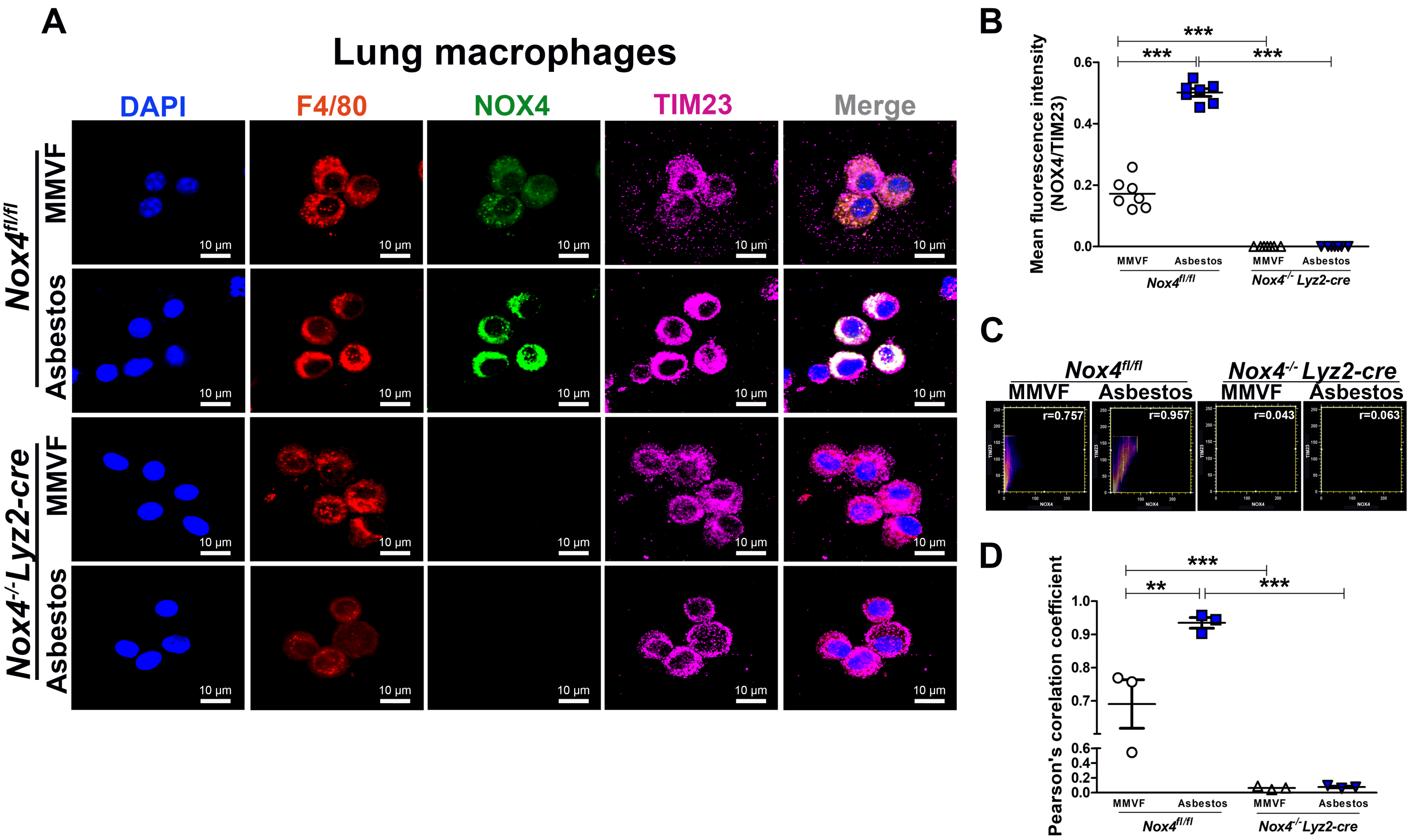

Supplement: Supporting Figure S3 [file mmc3.pdf]
