# Supplementary material for: NOX4–TIM23 interaction regulates NOX4 mitochondrial import and metabolic reprogramming
Source: J Biol Chem. 2023 Apr 10;299(5):104695. doi: 10.1016/j.jbc.2023.104695 (PMC10193017; doi:10.1016/j.jbc.2023.104695)
Supplement: Supporting Information [file mmc6.docx]

**Supplementary Data**

**NOX4-TIM23 interaction regulates NOX4 mitochondrial import and**

**metabolic reprogramming**

Authors: Jyotsana Pandey^1^, Jennifer L. Larson-Casey^1^, Mallikarjun H. Patil^1^, Rutwij Joshi^1^, Chun-sun Jiang, ^1^ Yong Zhou^1^, Chao He^1^, and A. Brent Carter^1,2*^

**Supplementary Figure 1. NOX4 overexpression augments interaction with TOM20.**

**A** Quantification of immunoblots for NOX4 overexpression**.** **B** Confocal microscopy shows co-localization of NOX4 and TOM20 in transfected macrophages with empty vector and hNOX4 Plasmid with and without asbestos (n=3). Representative images show NOX4 (green), TIM23 (red) and Nuclei (blue) scale bar 10 μm. **C** Bar graph for mean fluorescent intensity in macrophages (n=6). One way ANOVA followed by Tukey’s post hoc comparison test ***p < 0.001. **D** Giemsa staining of lung macrophages from *Nox4^fl/fl^* and *Nox4^-/-^Lyz2-cre* mice either treated with MMVF or Asbestos and **E** Cell population in BAL cells.

**Supplementary Figure 2. Transcriptional regulation of TIM23 by NOX4.**

**A** Immunoblots of macrophages treated with MG132 with and without asbestos. **B** NOX4 mRNA analysis in transfected macrophages with scrambled and siRNA *Nox4* with and without asbestos exposure (n=3). **C** TIM23 mRNA analysis in transfected macrophages with scrambled and siRNA *Nox4* with and without asbestos exposure (n=3). Data representative of mean (n=3) ± S.E.M. One way ANOVA with Tukey’s post hoc comparison test, ***p < 0.001.

**Supplementary Figure 3. NOX4 colocalizes with TIM23 in lung macrophages.**

**A**. Colocalization of NOX4 and TIM23 in lung macrophages of *Nox4****^fl/fl^*** mice and in macrophage from *Nox4****^-/-^*** *Lyz2-cre* with and without asbestos exposure (n=3). Representative images show NOX4 (green), TIM23 (pink), F4/80 (red) and Nuclei (blue) scale bar 10 μm**. B** Mean fluorescent intensity of interaction in lung macrophages (n=7). **C** Scatterplot of colocalization. **D** Pearson’s correlation coefficient (n=3).

**Supplementary Figure 4. NOX4 is required for OCR in lung macrophages.**

OCR tracing on the Seahorse XF96 bioanalyzer. *Nox4****^fl/fl^*** mice and *Nox4****^-/-^*** *Lyz2-cre* mice were exposed to MMVF, and asbestos for 21 days. Lung macrophages were subjected to OCR measurement **A** Basal OCR and **B** Maximum OCR. Data representative of mean (n=3) ± S.E.M. One way ANOVA with Tukey’s post hoc comparison test, ***p < 0.001.

**Supplementary Figure 5. Schematic summary of the study.** Asbestos exposure mediates direct interaction NOX4 and TIM23 in the mitochondria within the transmembrane domain of NOX4. This results in an increase in mitochondrial ROS and metabolic reprogramming to oxidative phosphorylation.
